# Integrating the Practical Robust Implementation and Sustainability Model With Best Practices in Clinical Decision Support Design: Implementation Science Approach

**DOI:** 10.2196/19676

**Published:** 2020-10-29

**Authors:** Katy E Trinkley, Michael G Kahn, Tellen D Bennett, Russell E Glasgow, Heather Haugen, David P Kao, Miranda E Kroehl, Chen-Tan Lin, Daniel C Malone, Daniel D Matlock

**Affiliations:** 1 Department of Clinical Pharmacy Skaggs School of Pharmacy and Pharmaceutical Sciences University of Colorado Anschutz Medical Campus Aurora, CO United States; 2 Department of Medicine University of Colorado Anschutz Medical Campus Aurora, CO United States; 3 Clinical Informatics University of Colorado Anschutz Medical Campus Aurora, CO United States; 4 Adult and Child Consortium for Outcomes Research and Delivery Science Aurora, CO United States; 5 Section of Informatics and Data Science Department of Pediatrics University of Colorado Anschutz Medical Campus Aurora, CO United States; 6 Department of Family Medicine School of Medicine University of Colorado Anschutz Medical Campus Aurora, CO United States; 7 Colorado Clinical and Translational Sciences Institute University of Colorado Anschutz Medical Campus Aurora, CO United States; 8 Charter Communications Corporation Greenwood Village, CO United States; 9 Department of Pharmacotherapy Skaggs College of Pharmacy University of Utah Salt Lake City, UT United States; 10 VA Eastern Colorado Geriatric Research Education and Clinical Center Aurora, CO United States

**Keywords:** clinical decision support, PRISM, implementation science

## Abstract

**Background:**

Clinical decision support (CDS) design best practices are intended to provide a narrative representation of factors that influence the success of CDS tools. However, they provide incomplete direction on evidence-based implementation principles.

**Objective:**

This study aims to describe an integrated approach toward applying an existing implementation science (IS) framework with CDS design best practices to improve the effectiveness, sustainability, and reproducibility of CDS implementations.

**Methods:**

We selected the Practical Robust Implementation and Sustainability Model (PRISM) IS framework. We identified areas where PRISM and CDS design best practices complemented each other and defined methods to address each. Lessons learned from applying these methods were then used to further refine the integrated approach.

**Results:**

Our integrated approach to applying PRISM with CDS design best practices consists of 5 key phases that iteratively interact and inform each other: multilevel stakeholder engagement, designing the CDS, design and usability testing, thoughtful deployment, and performance evaluation and maintenance. The approach is led by a dedicated implementation team that includes clinical informatics and analyst builder expertise.

**Conclusions:**

Integrating PRISM with CDS design best practices extends user-centered design and accounts for the multilevel, interacting, and dynamic factors that influence CDS implementation in health care. Integrating PRISM with CDS design best practices synthesizes the many known contextual factors that can influence the success of CDS tools, thereby enhancing the reproducibility and sustainability of CDS implementations. Others can adapt this approach to their situation to maximize and sustain CDS implementation success.

## Introduction

### Background

Clinical decision support (CDS) tools within electronic health records (EHRs) have led to some improvements in patient care [1-4] However, there are also numerous examples of CDS tools leading to low adoption or negative impact on outcomes. Up to 95% of CDS tools are dismissed [5], 52%-66% improve process outcomes such as appropriate drug selection, and only 21%-43% lead to improvements in clinical outcomes [6-9]. CDS design best practices may be a way to improve the impact of CDS tools [10,11]. As a framework, CDS design best practices are intended to provide a narrative representation of the key determinants that influence the success of CDS tools [12-18]. Textbox 1 provides a high-level summary of the best practices that include a user-centered design process. Initially established by experts in the field, retrospective studies support the potential benefit of applying CDS design best practices [13-16,19,20].

**Overview of clinical decision support design best practices and examples of how to address the best practice.**

Minimize alert fatigue

- Ensure accuracy and completeness of data and information recommended and used to formulate recommendation. Continual performance evaluation and end user feedback throughout implementation. Evaluations and feedback should be used to iteratively update the clinical decision support (CDS). CDS customization to fit the end user and institutional needs, including type (interruptive or passive), presence of a
  dismiss
  option, and frequency and timing of alert. Consideration of whether the CDS can support agenda setting

Support team-based care

- Comprehensive inclusion of care team members in which the CDS is tailored to the workflows and roles of each member

Fit within the end user’s workflow when considering other internal and external drivers

- Presentation of the CDS in such a way that it is available when needed, supports (versus impedes) end users, and human factors principles that makes it easy for them to synthesize and apply the information displayed (eg, visual cues such as size, position or color; prioritization; standardization). Flexibility to delay or defer CDS to another time or person can optimize workflow integration. Response options that reflect all possible patient situations (eg, other). Obtaining end user input on the design (user-centered design) with consideration of other internal and external factors such as national guidelines or value-based performance measures

Present pertinent and transparent information that supports and does not impair autonomy of decision-making

- Provide the rationale and supporting information (eg, references) on why the CDS is displayed so as to allow the end user to evaluate whether to apply the recommendation. Avoid giving a perception of shaming or use of insulting language

Make it easy, and incentivize users to follow the recommendation

- Provide actionable recommendations and functionality to save the end user time (eg, ability to place orders within display; links to review or update patient data). Provide a relative advantage to using the CDS (eg, peer or patient recognition for actions taken; save time).

CDS design best practices acknowledge the importance of implementation but do not provide a comprehensive framework or direction on evidence-based implementation principles. Some implementation science (IS) frameworks are available for health information technologies (health IT) [21-24]. However, there are no published reports that are comprehensive in providing direction for all stages of health IT or CDS implementation. A comprehensive IS framework could provide direction on how to account for field-specific nuances and contextual factors that influence the success of CDS tools [25]. Such an IS framework can allow health systems to proactively address the areas where things often break down in the process of adoption, implementation, and maintenance.

### Objectives

Here, we describe how to apply an existing IS framework to CDS to improve effectiveness, sustainability, and reproducibility. The specific objectives of this report are: (1) discuss how an IS framework can be integrated with CDS design best practices, (2) describe how to apply this integrated approach using illustrative case studies, and (3) discuss directions for future research and application of this integrated approach to CDS implementation.

## Methods

### Selection of an IS Framework

**Figure 1:**
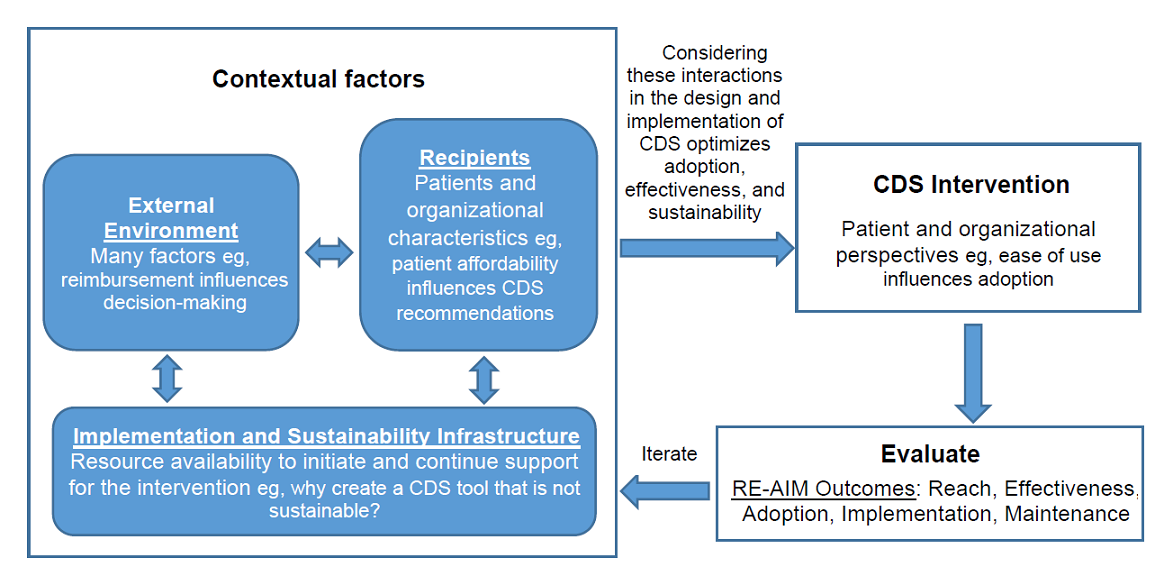
Domains of Practical Robust Implementation and Sustainability Model, their interactions, and how they influence clinical decision support. CDS: clinical decision support.

Although there are many IS frameworks [26], we selected the Practical Robust Implementation and Sustainability Model (PRISM) because it: (1) is a process, evaluation and determinants framework, (2) comprehensively addresses the interactions between the intervention and stakeholders and both organizational and external factors, (3) is directly tied to real-world, pragmatic implementation outcomes, and (4) is easy to use, thereby maximizing scalability [26-30]. As a process model, PRISM provides direction on how to address factors that influence implementation. As a determinant framework, PRISM makes it possible to reproduce the process for considering key factors that may influence implementation success [26]. As a comprehensive framework, PRISM considers all stages of implementation (preimplementation planning and design, implementation operations, postimplementation evaluation) and all groups or levels of influences within and external to the organization. As illustrated below, it is also compatible with CDS design best practices. Finally, PRISM builds on the Reach, Effectiveness, Adoption, Implementation, and Maintenance (RE-AIM) framework [31] for key implementation outcome measures. Textbox 2 describes the domains of PRISM and how they apply to CDS. Figure 1 depicts the domains of PRISM, their interactions, and how they influence CDS.

**PRISM domains and application to clinical decision support.**

**Intervention: organizational perspective includes leadership, management, clinicians, and frontline staff**

An intervention is more likely to be successful if:

- It is aligned with the organization’s mission and readiness for change
- The strength of evidence supporting the intervention is strong
- It addresses a barrier to or gap in health care
- It has been observed to be beneficial before a long-term commitment (observability, trialability, reversibility)
- It is simple and inexpensive

**Intervention: patient perspective**

An intervention is more likely to be successful if it is:

- Patient centered
- Simple and inexpensive
- Accessible to and understood by a wide variety of patients (cultural backgrounds, literacy, or numeracy levels)
- Addresses key patient concerns, not limited to clinical issues

**Recipients: organizational characteristics includes leadership, management, clinicians, and frontline staff**

Characteristics of the organization can impact the success of an intervention, such as financial health, tendency to take risks or deviate from the norm, and morale.

An intervention is more likely to be successful when:

- Management is supportive
- Goals are cohesive and clearly communicated across the organization
- Input is provided across all levels of the organization, including senior leadership, midlevel management, and pertinent frontline clinicians and staff

**Recipients: patient characteristics**

- Characteristics of patients can impact the success of an intervention, including socioeconomic factors such as affordability and access or transportation barriers to the intervention

**External environment**

- Factors outside of the organization can influence the organization, such as reporting on performance metrics (public face), policy, guidelines, and reimbursement issues

**Implementation and sustainability infrastructure**

- The implementation plan should be carefully crafted with a dedicated team for implementation, input from management and other stakeholders, and consideration of sustainability and dissemination from the beginning.
- Adequate resources and ongoing assessment or audit and feedback system should be in place

### Integration of the PRISM With Clinical Decision Support Design Best Practices

To account for the specific contextual factors of CDS in health care, we integrated PRISM with CDS design best practices, which we hereafter refer to as the PRISM/CDS best practice approach. Table 1 highlights areas where PRISM and CDS design best practices complement each other and the corresponding methods to address each. For example, clinician focus groups address both the PRISM domain of the *intervention* from the organizational perspective and CDS design best practice principle of *support team-based care*. Therefore, the focus group should include questions to understand who is involved in the specific care process, organizational barriers to prescribing, and whether the subject matter experts (SMEs) feel the strength of evidence is strong and clinically relevant. When performing the cross-walk between the 2 frameworks, there are situations where the individual principles and domains do not complement each other (empty cell in Table 1), which is appropriate. For example, when designing a CDS for clinicians to use, methods to address the best practice principle of *fit within end users workflow* would generally be unrelated to methods used to address the PRISM domain of *intervention* from the patient perspective. In general, instances in which cells are empty are because 1 framework focuses on the clinician’s perspective, the other focuses on the patient perspective, and there is no common area or overlap that integrates the 2 perspectives. Lessons learned from applying the methods outlined in Table 1 were then used to further refine the PRISM/CDS best practice approach.

**Table 1:** Practical Robust Implementation and Sustainability Model and clinical decision support design best practices: complementary areas and corresponding methods to address.

PRISM^a^ domains		Overarching CDS^b^ design best practice principles			
	Minimize alert fatigue	Support team-based care	Fit within the end user’s workflow when considering other internal and external drivers	Present pertinent and transparent information that supports and does not impair autonomy of decision making	Make it easy and incentivize users to follow the recommendation
Intervention: organizational perspective	EU^c^/clinician focus groups	EU/clinician focus groups	EU/clinician focus groups	EU/clinician design/usability testing	EU/clinician design/usability testing
	EU/clinician usability testing	EU/clinician design/usability testing	EU/clinician design/usability testing		
Intervention: patient perspective	N/A^d^	Patient focus groups and interviews	N/A	N/A	EU/patient focus groups and interviews
Recipients: organizational characteristics	N/A	EU/clinician focus groups	EU/clinician focus groups	EU/clinician design/usability testing	EU/clinician design/usability testing
			Clinician design/usability testing		
		EU^c^/clinician design/usability testing	Early engagement of leadership/ management		
Recipients: patient characteristic	N/A	EU/clinician focus groups	EU/clinician focus groups	N/A	N/A
		Patient focus groups			
External environment	N/A	N/A	Alignment with national payor and guideline metrics	N/A	Alignment with national payor and guideline metrics
Implementation and sustainability infrastructure	Scheduled performance evaluation and update	EU/clinician design/usability testing	EU/clinician design/usability testing	EU/clinician design/usability testing	EU/clinician design/usability testing including testing of training materials

^a^PRISM: Practical Robust Implementation and Sustainability Model.

^b^CDS: clinical decision support.

^c^EU: end user.

^d^N/A: Not applicable. Situations where the individual principles and domains do not complement each other.

### Case Study

Throughout this paper, we will refer to a CDS tool to improve the prescription of evidence-based beta blockers for patients with heart failure and reduced ejection fraction. CDS was deployed within the EHR of primary care practices across a large regional health system. The PRISM/CDS best practice approach was applied to the design and implementation of the CDS, as described in the 5 phases below. We note that the methods and results of a randomized controlled trial (RCT) evaluating the PRISM/CDS best practices approach are under review separately, but our focus here is on the application and integration of the approach, not outcome results.

## Results

### Overview

**Figure 2:**
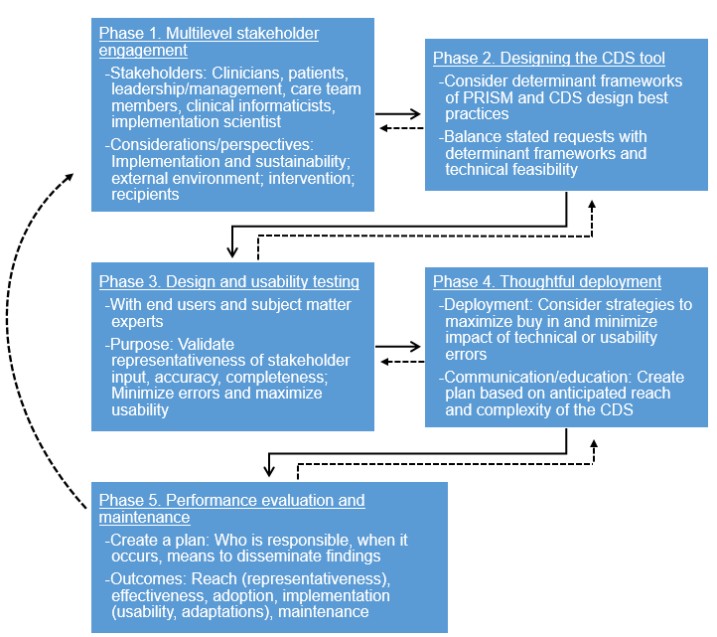
Phases of applying the Practical Robust Implementation and Sustainability Model to clinical decision support implementation. CDS: clinical decision support.

Our integrated approach to applying PRISM to CDS consists of 5 phases: (1) multilevel stakeholder engagement, (2) designing the CDS tool, (3) design and usability testing, (4) thoughtful deployment, and (5) performance evaluation and maintenance. Although there is some logical sequence to these phases, the process is not linear. The phases interact and iteratively inform each other. Figure 2 provides an overview of the phases, which emphasizes that the process is iterative and agile. Here, we describe each of the phases and the key determinants of implementation success from PRISM and CDS design best practices. These phases are intended to be adapted for each unique health system and CDS implementation.

### Phase 1. Multilevel Stakeholder Engagement

Multilevel stakeholder engagement is central to PRISM and extends the concept of user-centered design by incorporating stakeholders beyond the end user. PRISM emphasizes factors that can influence implementation success: perspectives of the patient and organization as well as characteristics of the recipients. Patient and organizational beliefs, technology support, and patient ability to afford medical care are some of the characteristics that can influence CDS implementation (Textbox 2 and Figure 1). Following PRISM, it is important to cultivate positive stakeholder perspectives of the CDS tool and identify recipient characteristics that can influence implementation success.

The types of stakeholders will vary for each CDS implementation and can range from a small homogenous group to large groups representing diverse disciplines and specialties. To build CDS tools that are trusted by end users, engaging the right stakeholders with the necessary skills is critical [32]. Stakeholders unique to health IT and CDS include health IT leadership or governance, clinical informaticists, and analyst builders. Of these stakeholders, a clinical informaticist and analyst builder should be part of a dedicated implementation team, whereas the role of health IT leadership or governance is generally to provide support and approval for implementation. The dedicated implementation team is a key component of the PRISM’s implementation and sustainability infrastructure. Often overlooked, clinical informaticists span across disciplines and are uniquely trained to empathize with clinicians, apply the determinants of effective CDS implementation, and balance what is asked for with what is technically possible [33-37]. Clinical informaticists should ideally have experience in IS or an IS expert should be engaged.

Gaining early support from health IT leadership is generally the first step, followed by input from SMEs and end users, and then formal governance approval. When patients are not the end users, their engagement is likely best reserved until after formal governance approval, just in case approval is not granted. The system’s health IT leadership assists in determining whether a particular CDS tool is generally aligned with the system’s priorities and whether resources are available to build the CDS tool. Health IT leadership can also provide direction regarding steps needed to secure necessary approval from the health system’s formal governance process. Although not all health systems have a formal health IT governance approval process, an increasing number do [38,39]. The organization and process for securing governance approval can vary greatly across health systems and can be complex; thus, early understanding is important. The formal governance approval process includes a review of CDS appropriateness from a workflow and safety perspective and considers potential overlapping or competing system-level initiatives.

SME and end user engagement is a dynamic process that often requires the clinical informaticist, leading the effort to iteratively engage each party until consensus on the design of the CDS tool is established. Obtaining input from SMEs is key to validating the clinical evidence, whereas input from end users is key to ensuring acceptance and practicality of the CDS tool. End user engagement is key to optimizing the workflow integration. The workflow and preferences of clinician end users can vary greatly across disciplines (eg, respiratory therapists, physicians) and practice settings (eg, acute care, dialysis center). The type of information elicited from patients or caregivers will depend on the type of CDS, notably whether the patient will be an end user or whether the CDS tool makes recommendations that might impact patient treatment decisions. Although patient-facing CDS tools are becoming more common, the majority are still clinician facing. When clinician facing, the line of questioning directed at patients or caregivers is on treatment priorities and values to ensure alignment with the CDS tool’s recommendations. When conducting focus groups or interviews with stakeholders, questions should be tailored to the situation [40] and informed by the determinants of effective implementation, which include CDS design best practices. The objectives of SMEs and end user engagement are to garner their support and define the general scope of the CDS tool. Such a written CDS scope can be used to facilitate the formal governance approval process. Textbox 3 describes our approach to engaging stakeholders.

**Phase 1 case study.**

Case study:

- For the beta-blocker CDS tool, the Chief Medical Information Officer (CMIO) was contacted at the start of the project. The CMIO provided support contingent on approval from other stakeholders and the formal governance process. To engage patient and clinician stakeholders in preimplementation design activities, we conducted focus groups rather than individual interviews to maximize the efficiency of resources and to facilitate idea generation among participants. However, we also sought to elicit individual patient and clinician thoughts.
- Clinicians, after the open-ended group discussion regarding needs and preferences for a CDS tool, were asked to individually design on paper their ideal CDS [41], which provided valuable insights. For example, most clinicians expressed strong dislike for interruptive CDS during group discussion, but many individually described their ideal CDS as being interruptive. The discordance may be the result of peer influence or reflect differences between their preferences and their perceptions of what is most effective. These findings led to an interruptive CDS tool [41].
- In our case, the CDS tool was clinician facing; thus, the goal of the patient focus groups was to evaluate the factors that influence patients’ decisions to take heart failure medications. The focus groups occurred early in the design process to ensure that the prototypes were driven by patient-centered factors. We found that patient values and preferences for heart failure medications aligned with clinical guideline recommendations that prioritize benefits over risks, cost, and the inconvenience of taking medications. These findings provided reassurance that we were designing a CDS tool that reflected patient priorities.

### Phase 2. Designing the CDS Tool

Following PRISM, stakeholder input is used to design the intervention to maximize the recipient’s perceived value and ensure sufficient support infrastructure (Textbox 2). Such interventions are more likely to be successful and sustained over time. PRISM emphasizes the importance of designing interventions with sustainability in mind.

Once there is general agreement or saturation of ideas [42] from the stakeholders, this information is used to draft the build scope of the CDS tool. The CDS scope includes an idea of the user interface (UI) content, workflow integration, and format (eg, interruptive alert versus passive, mobile app) for interfacing with end users. The clinical informaticist drafts the build scope based on what is clinically relevant, the stated workflow needs and preferences of the stakeholders, organizational priorities, and patient-centered considerations. Aligned with the learning health care system and agile design principles, the first CDS build scope is not intended to be perfect, rather a first iteration [43]. However, every iteration must be vetted to *do no harm*.

Stakeholders suggest many ideas. If they are all followed, the CDS UI may not be intuitive because too much information is presented. It is the role of the clinical informaticist to identify the most salient stakeholder requests and determine the appropriate balance between user-centered design and other determinants of implementation success outlined by PRISM and CDS design best practices. Finding this balance is challenging and varies for each situation. Textbox 4 provides an example of a situation in which stakeholder requests were balanced with CDS design best practices. CDS in health care is fraught with nuances, and design decisions require thoughtful consideration of multiple dynamic and interacting contextual factors. The clinical informaticist also liaises between the stakeholders and build analysts (or they may be the builder), advocating for the stakeholders as appropriate and creatively adapting to the constraints of the EHR technical infrastructure, standards, and local resources to ultimately fit within the clinical workflows. Standards can include norms around the appearance and presentation of CDS tools within the organization or external technical standards to promote interoperability, such as Health Level 7 and use of standardized vocabularies such as SNOMED (Systematized Nomenclature of Medicine) to classify diagnoses. An important consideration is the most appropriate technical integration format for the CDS tool, which could include the use of native EHR software, web services, or Substitutable Medical Apps Reusable Technology on Fast Healthcare Interoperability Resources applications. Each institution’s EHR offers a unique set of options to integrate CDS tools natively or interface them with external software. A clinical informaticist will be abreast of what technical integration options each institution has available and guides the design accordingly.

**Phase 2 case study.**

Case study:

When designing the beta-blocker CDS tool, some stakeholders requested that a complete list of patient medications be included in the UI. However, most patients with heart failure are on many medications. Compliance with that request would have significantly decreased intuitiveness of the CDS tool. Balancing this request with CDS design best practices, it was decided not to include a complete medication list within the UI. However, this decision was difficult, given that this was a consistent request from stakeholders and the information influenced their decision-making. Without inclusion of this information in the UI, clinicians who did not recall concurrent medications would have to leave the CDS tool and search elsewhere in the EHR for the information, which decreases the relative advantage of the CDS tool. Later iterations of the CDS tool will consider creative ways to integrate medications with the CDS tool (eg, info button functionality).

### Phase 3. Design and Usability Testing

Following PRISM, at each stage of stakeholder engagement, new insights are learned. As CDS development activities evolve to testing, the format and nature of stakeholder engagement changes, but patient safety issues are always considered. Aligned with PRISM, design and usability testing aim to ensure that the CDS tool is designed well and simple to use. Design testing includes both SMEs and end users, whereas SMEs may be conditionally included in usability testing if their inclusion optimizes general buy-in. Design testing does not necessarily require build completion. However, before usability testing begins, the build should be complete within EHR testing environments and thoroughly tested to ensure that there are no errors. The resolution of build errors maximizes the focus during usability testing on optimizing the end user experience. When possible, the use of >1 UI during design and usability testing can enrich preimplementation design activities [44].

Testing can be completed in a variety of ways. Exemplars of design and usability testing can be found outside of health care [45], but for a variety of reasons have proven difficult to apply in health care. Therefore, here we describe an approach that can be practically applied in health care. Testing in actual clinical scenarios is not always possible in health care; thus, we propose design testing in which static screenshots of the CDS UI are shared with stakeholders, and usability testing with simulated patient scenarios followed by open-ended discussion. Design testing serves to validate whether the stated needs and preferences of stakeholders are accurately represented in the UI. During design testing, the UI is iteratively updated before commencement of the more resource-intensive usability testing.

During usability testing, proctored simulation coupled with the *think aloud* protocol [46] and open-ended discussion can serve to inform educational materials, identify usability issues before going live, and identify additional areas for improvement, such as unintended consequences. Using the *think aloud* protocol during usability testing simulations can help identify barriers to following the CDS tool. Such barriers can be elaborated on during the open-ended discussion and, when actionable, the CDS tool can be redesigned to address the barrier. Proctored, simulated patient scenarios may not always be feasible given time or other resource constraints. Less resource-intensive usability testing methods could consist of asking end users to test the CDS tool remotely in EHR testing environments at their convenience and reporting back electronically with any feedback.

During design and usability testing, it is important to get end users in the mindset of their clinical workflow and consider (1) all members of team-based care, (2) factors that would impede or aid their workflow (cause alert fatigue versus fit into workflow), (3) whether the CDS tool supports their ability to make an informed decision, (4) whether it makes it easy to take (or incentivize) action, and (5) potential barriers to following the CDS tool’s recommendation, especially over time. The determinants of effective implementation and sustainability from PRISM (Textbox 2) and CDS design best practices (Textbox 1) should inform specific questions to be asked during testing. Textbox 5 describes our approach to design and usability testing.

**Phase 3 case study.**

Case study:

For our beta-blocker CDS tool, design testing occurred via email. Stakeholders were asked for input with specific questions, which we found to be effective and efficient. During usability testing, we incorporated our educational handout as part of the simulations, which resulted in substantial revisions to the handout as a result of end user feedback. Furthermore, during usability testing, we discovered that many clinicians were unaware that respiratory disease was not a contraindication to beta blockers; thus, a statement to address this misconception was added to the UI. Multimedia Appendix 1 provides examples of feedback from stakeholders during design and usability testing and reasons for or not incorporating into the CDS design.

### Phase 4. Thoughtful Deployment

The implementation and sustainability infrastructure domain of PRISM considers practical measures to facilitate the ease of workflow integration. Adoption is improved when the necessary support to use the intervention is provided, and there is a means to quickly resolve any unintended consequences. Thoughtful introduction into clinical workflows is imperative for the adoption of CDS tools. Deployment should consider how to move the CDS tool into actual clinical workflows and how to communicate the change with end users. Re-engagement with clinical leadership before deployment is also key, especially in situations in which the preimplementation process occurred over a long period of time or for health systems undergoing certain changes.

The decision to implement the CDS tool in a pilot cohort or begin with widespread deployment is an important consideration to resolve any remaining usability or technical errors and to facilitate buy-in. In some instances, it is prudent to begin with a pilot group of users, which may include one clinical department or group of users across departments. The decision to deploy the CDS tool on a large or small scale initially should be informed by the anticipated frequency of exposure, acuity of the clinical situation, and workflow disruption of the CDS tool. In the case of a CDS tool with infrequent end user exposure, widespread dissemination can serve as a natural pilot when appropriately monitored. Implementing CDS in a pilot fashion can help to bolster buy-in and to discover unintended issues before widespread roll-out.

With every CDS go-live, some communication with end users is needed but the extent should vary based on the complexity of the CDS tool and the anticipated frequency of end user exposure. When exposure to a CDS tool is infrequent or highly intuitive, end user education may not be necessary. However, an interruptive CDS tool that recommends discontinuation of nonsteroidal anti-inflammatory drugs in patients with cardiovascular disease would likely require some education of end users because of its alert frequency and obtrusiveness. In the latter example, end user education may provide a more detailed explanation of why this new tool is being implemented and what the response options provided within the UI mean. For example, the response options may include *never appropriate* without space to explain within the CDS UI that selecting this option will suppress the CDS tool forever for all clinicians. It would be helpful to share such information in the educational material. Many clinicians wish to know the consequences of their actions in response to a CDS tool. Communication plans should be informed by the given health system’s standard processes and adapted based on the type of CDS and anticipated impact on the end user. Textbox 6 describes key experiences from our deployment.

**Phase 4 case study.**

Case study:

- For the beta-blocker CDS tool, re-engagement with the leadership of the clinical practices before deployment was pivotal. During our preimplementation planning process, the health system acquired several outpatient practice groups, which resulted in changes in priorities and approaches to decision support. At the beginning of the preimplementation planning stage, we secured leadership approval to deploy the CDS tool across all practices; however, given the changes that occurred, this approval was no longer applicable. Therefore, we needed to solicit approval for deployment from individual practices. Our experience emphasizes the need to maintain frequent engagement with leadership and a nonlinear approach to CDS implementation.
- When soliciting approval from individual practices, we piloted the CDS tool in 2 of the largest practices. These 2 pilot practices expressed early support for the CDS tool. When soliciting approval from other practices, they found it reassuring to know the tool was already accepted by their peers and being tested.

### Phase 5. Performance Evaluation and Maintenance

PRISM includes the RE-AIM evaluation framework and outcomes. RE-AIM captures a broad and balanced evaluation of the nuances and pragmatic nature of implementation in clinical workflows [31]. Such a multilevel IS framework assesses the representativeness of participants, the extent to which the intervention needs to be adapted, and its sustainability [27-30]. Adaptions to interventions should be anticipated and evaluated. RE-AIM also encourages continuous evaluation and dissemination of findings to promote observability and thereby optimize implementation and sustainability. Table 2 provides an example of how RE-AIM can be applied to CDS. For example, changes in clinical outcomes are difficult to associate with CDS tools; thus, effectiveness is often measured as a change in behavior. Other instruments and tools can also be used to evaluate CDS tools. The usability of CDS tools can be evaluated using the validated System Usability Scale (SUS) [47].

**Table 2:** Reach, Effectiveness, Adoption, Implementation, and Maintenance evaluation framework applied to clinical decision support.

RE-AIM^a^ domain	Described	Potential CDS^b^ outcome measures
Reach (individual level)	Proportion and representativeness of those impacted by the intervention (and reasons for these results)	Number of patients the CDS tool fired for divided by the number of patients the CDS tool should have fired for Characteristics of each group in numerator and denominator Investigation of reasons not fired
Effectiveness (individual level)	Impact of the intervention, including heterogeneity across subgroups and any negative outcomes (and reasons for these results)	Number of patients the CDS tool changed care for divided by the number of patients the CDS tool fired for Characteristics of each group in numerator and denominator Reasons care did or did not change Number and type of unintended or negative outcomes
Adoption (setting and staff at multiple levels)	Proportion and representativeness of those accepting or using the intervention At levels of health systems, departments, and individuals (and reasons for these results)	Number of clinicians who responded^c^ to the CDS tool (did not outright dismiss) divided by the number of clinicians the CDS tool fired for Number of patients who the CDS fired for that were not outright dismissed divided by the number of patients the CDS tool fired for Number of practices, setting or clinicians participating divided by the number invited Characteristics of each group in the numerators and denominators above Reasons for or not to participate or dismiss
Implementation (setting and staff at multiple levels)	Fidelity of the intervention and implementation strategy Adaptations Burden of delivery, including costs	Adaptation: number and type of changes to the CDS build or workflow integration after deployment Usability of the CDS tool (eg, SUS^d^) Interviews on experience and adaptations Cost of implementing (eg, time, resources)
Maintenance (individual level and setting and staff at multiple levels)	Long-term effects of the intervention and extent the intervention becomes a routine part of care	Long-term outcomes (eg, change in mortality) Sustained workflow integration and effectiveness Interviews on intended or actual sustainment or further modification

^a^RE-AIM: Reach, Effectiveness, Adoption, Implementation, and Maintenance framework.

^b^CDS: clinical decision support.

^c^Technically in PRISM or RE-AIM, adoption is defined as only initial agreement to participate in (or be trained in) a program. In this paper, it will be defined as above to be consistent with how this term is used in informatics and to reflect the fact that end users do not always have the choice to interface with a CDS tool.

^d^SUS: System Usability Scale.

CDS tools are critical components of the learning health care system and should be regularly evaluated for performance and safety. Performance and safety evaluations are necessary for CDS maintenance and assist in minimizing alert fatigue. Evaluations should be scheduled with defined procedures of who is responsible, what the evaluation entails, and when it occurs. What is evaluated should be informed by operational leadership and influenced by external drivers, such as regulatory requirements and pay for performance metrics. The evaluation can also be informed by input from stakeholders regarding unintended consequences. For example, stakeholders might express concern that a CDS tool may lead to an increased risk of bradycardia; thus, this is an outcome that should be monitored.

Evaluations should not be limited to 1 instance and should lead to action when appropriate. CDS implementation should be an iterative, thoughtful, and continuous improvement process. Proactively seeking end user input during and post exposure also demonstrates commitment to improvement. Findings from evaluations should be disseminated to stakeholders, including appropriate levels of leadership, SMEs, and end users. Appropriate levels of leadership should vary based on the specific CDS tool and do not necessarily require notification at the chief executive level. Communication with individual SMEs and end users may be at the discretion of and via their direct leaders but should be offered at a minimum. This level of transparent communication can promote trust, improve adoption, and reduce the culture of negativity that currently exists around CDS and EHRs [48]. Textbox 7 describes key findings from our evaluation approach.

**Phase 5 case study.**

Case study:

- We monitored clinician responses and feedback to the beta-blocker CDS tool weekly to screen for unintended consequences and errors. After 1 month of deployment, a clinician alerted us to an error in the build of our CDS tool, which we were able to resolve quickly. Had our CDS tool fired more frequently, we would likely have identified this error during our 2-week pilot. Such errors are common when a CDS tool is deployed, reinforcing the importance of ongoing monitoring and clear lines of communication between the informatics team and the end user.
- We also conducted brief structured interviews with clinicians exposed to the CDS tool. Together with completion of the SUS survey, the interviews provided valuable open-ended feedback that was used to refine both the CDS tool and our PRISM/CDS best practices approach. Key discoveries made during the interviews were the importance of carefully considering whether to include a dismiss button and avoiding a sense of shaming or disrespect. These discoveries were explicitly added as determinants of implementation success within CDS design best practices in Textbox 1.

## Discussion

### Principal Findings

The PRISM/CDS best practices approach accounts for the multilevel interactions and dynamic factors that influence CDS implementation in health care. IS frameworks make social science more replicable and, by adding the context of CDS to PRISM, the reproducibility and sustainability of CDS implementations should be enhanced. *Integrating PRISM with CDS design best practices synthesizes the many known contextual factors that can influence the success of CDS implementation*, thereby elevating the experience from implementation to IS. This approach can be adapted for other health systems and CDS tools and used to guide resource allocation in a manner that optimizes CDS implementation success.

However, an effective approach may not be optimally efficient. When applying PRISM to CDS, resources include the time and availability of skilled personnel. Especially during the preimplementation period, this approach can require resources that may not be available at every health system or be appropriate allocation to every CDS instance. It may not be appropriate to allocate extensive resources to implementation efforts for CDS tools that are minimally invasive (eg, infobuttons) or address infrequent care gaps or low severity clinical situations. When resources are limited or the situation does not justify the allocation of full resources, stakeholder engagement may be abbreviated. Abbreviated stakeholder engagement may be limited to a smaller sample, representing a few representatives from each stakeholder group. Irrespective of resource availability, stakeholders should include representation from SMEs and end users. If allowed by resources, greater representation reaching saturation and general agreement from SMEs and end users is ideal.

The results of an RCT demonstrating the positive effect of the PRISM/CDS best practice approach on prescribing for heart failure in primary care is currently under review for publication. However, there are several limitations of the PRISM/CDS best practice approach. Although our study team represents diverse expertise, including informatics and IS, the approach was created based on our knowledge and experiences. Inclusion of additional expertise and experiences across other clinical contexts and situations would likely refine the PRISM/CDS best practices approach. Our approach also relies on one of many IS frameworks and existing CDS design best practices. Other frameworks may have different advantages or disadvantages when applied to CDS. As CDS design best practices and technical capabilities evolve, the PRISM/CDS best practices approach will need to adapt. Further, the PRISM/CDS best practices need to be adapted based on a given institution’s available resources and skilled personnel. Despite these limitations, the PRISM/CDS best practices approach provides a basis for advancing the science of CDS implementation.

Future research is needed to apply PRISM to additional real-world CDS implementations to capture all contextual factors and understand its impact on CDS adoption and effectiveness. Although the PRISM/CDS best practices approach was created for adaptation across any health care setting, it was refined within primary care settings across a large health care system. Future research should explore the application of PRISM to diverse CDS formats (eg, mobile apps) in a variety of patient care situations and practice settings of different sizes and identify means to refine the model to maximize effectiveness and efficiency. Use of the PRISM/CDS best practices approach should be documented in terms of its costs and benefits relative to other approaches. A key issue for both PRISM and CDS design best practices is the degree of iteration and number of cycles required for a given implementation. Efforts to improve the efficiency of the PRISM/CDS best practices approach are needed and may include integration with principles of rapid prototyping or tailoring the approach based on the severity of the clinical situation or the anticipated reach or workflow interruption of a given CDS tool. This tradeoff between resource allocation and benefits will have to be evaluated for every setting and clinical situation. Further, given that many of the contextual issues related to CDS are applicable to other types of health IT solutions, the applicability of this integrated approach to health IT beyond CDS should also be explored. Although the PRISM/CDS best practices approach provides guidance on evaluation, future research should consider how the approach can be expanded to include a systematic and standardized knowledge management process to evaluate and update CDS tools.

### Conclusions

We described an approach for applying PRISM to design, implement, and evaluate CDS tools that are integrated with CDS design best practices. Others are encouraged to adapt this approach to their situation to maximize CDS implementation success. This approach considers the many dynamic and interacting contextual factors that influence CDS implementation success and sustainability in health care and suggests specific methods for designing and implementing CDS. Informed by an evidence-based IS framework, such an approach is foundational to maximizing the success of CDS implementation and the necessary platform from which cutting-edge innovations in CDS can be created to significantly improve and sustain health care outcomes.

## Appendix

Multimedia Appendix 1 Summary of constructive feedback received during design and usability testing.

## Abbreviations

CDS: clinical decision support
CTSA: Clinical and Translational Science Award
EHR: electronic health record
Health IT: health information technology
IS: implementation science
PRISM: Practical Robust Implementation and Sustainability Model
RE-AIM: Reach, Effectiveness, Adoption, Implementation, and Maintenance
SME: subject matter expert
SUS: System Usability Scale
UI: user interface
