# Supplementary material for: Integrating the Practical Robust Implementation and Sustainability Model With Best Practices in Clinical Decision Support Design: Implementation Science Approach
Source: J Med Internet Res. 2020 Oct 29;22(10):e19676. doi: 10.2196/19676 (PMC7661234; doi:10.2196/19676)
Supplement: Multimedia Appendix 1 [file jmir_v22i10e19676_app1.docx]

**Multimedia Appendix 1.** Summary of constructive feedback received during design and usability testing.

|  |
| --- |

| **Timing of suggestion** | **Suggestion** | **Incorporated into CDS design and why?** |
| --- | --- | --- |
| Design testing | Automate association of beta blocker order with visit billing diagnosis | No, not possible given technical constraints |
|  | Change order of information presented in the UI | Yes, when possible within technical constraints to improve flow |
|  | State that the recommendation was supported by the head of cardiology | No, this was not a consensus |
|  | Present within the UI all factors that led the CDS to alert | No, this was not a consensus |
|  | Automatically discontinue non-evidence based beta blockers when an evidence based beta blocker is ordered | No, not possible given technical constraints |
|  | Change language stating absolute risk reduction to be a range | Yes, easy to change and appeals to different end users |

| Usability testing | Change acknowledge reason to be a drop down list | No, not possible given technical constraints |
| --- | --- | --- |
|  | Add statement addressing interaction between hypoglycemia and beta blockers | No, this was the minority and needed to keep UI concise |
|  | Add statement addressing interaction between asthma/COPD and beta blockers | Yes, this was a common request |
|  | Note that metoprolol tartrate is not evidence based | Yes, this was a common request |
|  | Default beta blocker orders to include 3 month supply each and 3 refills | No, this was the minority |
|  | Default beta blocker orders to include 1 month supply and 1 refill | No, this was the minority |
|  | Default beta blocker orders to include 3 month supply and 1 refill | Yes, this was the majority and a good balance to promote patient follow up and titration with patient safety concern of running out of meds |
|  | Make the font for the ejection fraction value larger and red | Yes, the majority |
|  | Preface “discontinue other beta blockers” with “please note” | No, can be more concise |
|  | Preface “discontinue other beta blockers” with “reminder” | Yes, more succinct than other suggestions and generally felt more respectful |
|  | Add instructions to titrate up after initiating | No, too much information and detracts from immediate need of starting |
|  | Associate medication orders with visit billing diagnosis | No, not possible given technical constraints |
|  | Associate medication order with indication | Yes, improves workflow |
|  | Link out to medication list | No, it would close the current UI and open a new window and the user would not be able to return to the UI |
|  | Add list of current medications or heart failure medications | No, too much information and would prevent the UI from being seen on one screen without scrolling |
|  | Include option to hover and see current medication list | No, not possible given current technical constraints |
